# Cancer Care in Afghanistan: Perspectives on Health Services Under the Taliban Regime

**DOI:** 10.1200/GO.23.00358

**Published:** 2023-11-16

**Authors:** Alaha Nasari, Amina Surosh Nasari, Sammer Marzouk, Edward Christopher Dee, Faramarz Jahanbeen

**Affiliations:** Alaha Nasari, BA, Harvard University, Cambridge, MA; Amina Surosh Nasari, MD, CUNY School of Medicine, New York, NY; Sammer Marzouk, MA, Harvard University, Cambridge, MA; Edward Christopher Dee, MD, Department of Radiation Oncology, Memorial Sloan Kettering Cancer Center, New York, NY; and Faramarz Jahanbeen, MD, MBA, MPH, Harvard T.H. Chan School of Public Health, Boston, MA

## Abstract

Cancer landscape in Afghanistan continues to suffer under Taliban—global support is essential.

## TO THE EDITOR:

In the wake of Afghanistan's change in leadership in 2021, a closer examination of the nation's health system reveals a persistent struggle for access to health care services. The recently updated Afghanistan Humanitarian Response Plan for 2023, published by the United Nations (UN) Office for the Coordination of Humanitarian Affairs, has presented a deeply concerning escalation in the magnitude of the ongoing humanitarian crisis. According to the plan's findings, the number of individuals in need of humanitarian aid has surged to a staggering 28.8 million, marking a sharp rise from the previous count of 18.4 million recorded before August 2021.^1^ In response, the WHO recently administered an Alert emphasizing the need to increase investments in health care service delivery across Afghanistan. This focus is particularly critical in underserved regions, where the health care infrastructure grapples with insufficient funding, limited medical supplies, and unskilled personnel.^2^ As the health system in Afghanistan continues to deteriorate, there is a growing focus on the critical state of cancer care.

The provision of cancer care is currently centered around a single cancer ward within Kabul's Jamhuriat Hospital. This specialized facility offers a range of cancer care services, including prevention, screening, chemotherapy, surgical oncology, and palliative care. The operational capacity of the ward, however, is severely limited, accommodating just 30 beds each for medical oncology, surgical oncology, and day care services. This forces most patients to resort to makeshift accommodations within the hospital corridors, enduring lengthy waits in hopes of securing an available bed. While regional hospitals in Mazar-e Sharif and Herat offer therapeutic interventions, they lack essential screening and palliative care services. Radiotherapy remains entirely absent within Afghanistan's cancer landscape. As a result, individuals with the means to do so cross the borders into Pakistan or Iran, seeking cancer treatments that have not yet established a presence within the country's domestic health care infrastructure.^3^

The most recent accessible cancer data for Afghanistan dates back to 2020, with one analysis revealing that the most prevalent cancers among both sexes in Afghanistan were breast (14.3% of cases), stomach (7.8%), lung (6.6%), cervix uteri (5.4%), and colorectum (4.9%).^4^ Afghanistan's Ministry of Public Health, now under Taliban control, reported earlier this year a rise in annual cancer diagnoses compared with previous years. According to Sharafat Zaman, a spokesperson for the Taliban, this trend is attributed to factors such as chemical usage and the diverse array of weaponry used within the country.^5^

Faramarz Jahanbeen, MD, MBA, MPH, who served as the Technical Manager for the Health Management Information System at Particip GmbH in Kabul, draws attention to the national and global ramifications of Afghanistan's underdeveloped cancer care infrastructure, “Without accurate methods for data collection, healthcare providers and policymakers do not have the insights needed to make informed decisions regarding cancer prevention, diagnosis, and treatment. This impedes our ability to identify trends, assess the effectiveness of interventions, and tailor health care strategies to the needs of the population. It also impedes Afghanistan's participation in global research efforts and collaborations, limiting our contribution to advancements in cancer on the international scale.” Dr Jahanbeen emphasized the need to channel resources into cancer data infrastructure as a means of fortifying Afghanistan's cancer response.

The absence of cancer data infrastructure is further compounded by a shortage of health care workers to address the needs of patients with cancer. Mastora Shafahi, MS, General Director and Founder of the Education and Health Organization for Afghanistan's Women, a women's rights nonprofit organization in Kabul, describes, “Working under the Taliban is not easy—doctors fear for their lives. There is fear even to request medicine. Doctors, prioritizing their safety, will seize the opportunity to seek refuge in other countries. The remaining doctors mainly consist of those who align with the Taliban's positions. Currently, there is an especially urgent need for oncologists—hospitals have even offered higher salaries for this role. In most situations, hospitals will resort to nurses to care for cancer patients.”

Access to health care is exacerbated by a shortage of female health care workers, a situation that is anticipated to worsen after the recent regulatory measures implemented by the Taliban at the end of 2022. These measures involve the prohibition of women from pursuing higher education or working for international or national nongovernmental organizations (NGOs). This has caused disruption in the operation of global health NGOs, which previously occupied a central role in the nation's public-private health care delivery structure.^6^ The consequences for female patients have been detrimental. “Women are not allowed to go to the clinic unless they are on the brink of death. If female doctors cannot work, then who can see our female patients? Other restrictions, even the mandatory female dress code, endanger the safety of women. The head-to-toe covering carries germs into the hospital,” Mastora shares.

What should be done? After all, the WHO designation of Alert only finds meaning when words are matched by efforts to improve access to care.

First, the onus falls on Afghanistan's leadership, regardless of ethnic or political affiliation, to prioritize improving access to cancer care. The Taliban, who oversee medical facilities, must create a secure and conducive environment that enables health care professionals to provide essential care to patients with cancer. This ensures both the ability of physicians to request support and the ability of patients to access care without fear. The Taliban must also allocate resources and funds to rebuild and update health care infrastructure, including cancer treatment centers, hospitals, and clinics. Facilities must be equipped with the necessary medical equipment, technology, and skilled health care professionals.

The advancement of cancer care facilities is contingent on the support of international donors. In the face of ongoing conflict and limited domestic funds, the Afghan health care system is strained. As of the last year, at least half a dozen prominent foreign aid organizations announced a temporary halt to their activities in Afghanistan.^7^ International medical organizations, NGOs, and governments must partner with Afghan health care institutions to provide technical assistance and expertise. The international community can also provide immediate humanitarian aid and medical supplies to address the immediate challenges faced by health care providers and patients with cancer.

Within the country, the Taliban should seek guidance from health care professionals to direct their partnerships with international entities. Medical experts can contribute to health policy developments by assessing cancer data and statistics. The Taliban must therefore support the advancement of the national cancer registry to collect accurate data on cancer prevalence, types, and treatment outcomes—critical information for planning effective interventions. This will contribute to developing a long-term plan for a sustainable health care system that involves continuous training, infrastructure maintenance, and health care delivery reforms. Specifically, training programs should be implemented to improve the skills of health care professionals in the areas of cancer diagnosis, treatment, and care.

To fully understand the care disparities in Afghanistan, and the barriers that facilitate these disparities, a sociocultural approach must be adopted. While collaborating with pharmaceutical companies, NGOs, and international organizations is certainly important to ensure a steady and affordable supply of essential cancer medications, understanding access to these medications involves delving into the social, structural, political, and historical determinants of health. In recognition of the UN 2030 Sustainable Development Goals and the 2017 WHO Cancer Resolution, recent work demonstrates support for incorporating these conceptual frameworks at the highest levels of political discourse to understand country-specific availability, accessibility, and affordability of cancer medications.^8^ In the context of Afghanistan, political instability, gender norms, financial constraints, geographical challenges, and limited public awareness about medical services are only a few examples of why barriers exist.^9^ Addressing the root causes of barriers allows us to deconstruct the existing system and reconstruct it in a way that prevents the recurrence of these barriers.

The Taliban transition to power has introduced challenges that only exacerbate an already fragile and undersized health system. In the midst of these challenges, Afghanistan's cancer landscape continues to suffer, and the quest for a solution continues to endure prolonged delays. Only through the cooperation of Taliban officials, the support of the international community, the insights of medical professionals and data collection, and a sociocultural approach to health can a solution be realized.
