# Erratum: Cancer Care in Afghanistan: Perspectives on Health Services Under the Taliban Regime

**DOI:** 10.1200/GO.23.00450

**Published:** 2024-01-11

The article by Nasari et al entitled “Cancer Care in Afghanistan: Perspectives on Health Services Under the Taliban Regime” (*JCO Global Oncology* 10.1200/GO.23.00358) was published online November 16, 2023, with an error.

An Acknowledgment to Dr Maihan Abdullah was incorrectly included.

This has been corrected as of January 11, 2024. We apologize for the error.

